# ADAR2 deficiency ameliorates non‐alcoholic fatty liver disease and muscle atrophy through modulating serum amyloid A1

**DOI:** 10.1002/jcsm.13460

**Published:** 2024-03-27

**Authors:** Mei‐Lang Kung, Tai‐Hua Yang, Chia‐Chi Lin, Jia‐Yun Ho, Tzu‐Chi Hung, Chih‐Hsiang Chang, Kuan‐Wen Huang, Chien‐Chin Chen, Yun‐Wen Chen

**Affiliations:** ^1^ Department of Medical Education and Research Kaohsiung Veterans General Hospital Kaohsiung Taiwan; ^2^ Department of Biomedical Engineering College of Engineering, National Cheng Kung University Tainan Taiwan; ^3^ Department of Orthopedic Surgery National Cheng Kung University Hospital, College of Medicine, National Cheng Kung University Tainan Taiwan; ^4^ Department of Pharmacology College of Medicine, National Cheng Kung University Tainan Taiwan; ^5^ Department of Pathology Ditmanson Medical Foundation Chia‐Yi Christian Hospital Chiayi Taiwan; ^6^ Department of Cosmetic Science Chia Nan University of Pharmacy and Science Tainan Taiwan; ^7^ Ph.D. Program in Translational Medicine, Rong Hsing Research Center for Translational Medicine National Chung Hsing University Taichung Taiwan; ^8^ Department of Biotechnology and Bioindustry Sciences College of Bioscience and Biotechnology, National Cheng Kung University Tainan Taiwan

**Keywords:** ADAR2, diabetes, inflammation, muscle atrophy, NAFLD, SAA1

## Abstract

**Background:**

Non‐alcoholic fatty liver disease (NAFLD) is the most common cause of chronic liver disease worldwide. Sarcopenia is a syndrome characterized by progressive and generalized loss of skeletal muscle mass and strength, which is commonly associated with NAFLD. Adenosine‐to‐inosine editing, catalysed by adenosine deaminase acting on RNA (ADAR), is an important post‐transcriptional modification of genome‐encoded RNA transcripts. Three ADAR gene family members, including ADAR1, ADAR2 and ADAR3, have been identified. However, the functional role of ADAR2 in obesity‐associated NAFLD and sarcopenia remains unclear.

**Methods:**

ADAR2^+/+^/GluR‐B^R/R^ mice (wild type [WT]) and ADAR2^−/−^/GluR‐B^R/R^ mice (ADAR2 knockout [KO]) were subjected to feeding with standard chow or high‐fat diet (HFD) for 20 weeks at the age of 5 weeks. The metabolic parameters, hepatic lipid droplet, grip strength test, rotarod test, muscle weight, fibre cross‐sectional area (CSA), fibre types and protein associated with protein degradation were examined. Systemic and local tissues serum amyloid A1 (SAA1) were measured. The effects of SAA1 on C2C12 myotube atrophy were investigated.

**Results:**

ADAR2 KO mice fed with HFD exhibited lower body weight (−7.7%, *P* < 0.05), lower liver tissue weight (−20%, *P* < 0.05), reduced liver lipid droplets in concert with a decrease in hepatic triglyceride content (−24%, *P* < 0.001) and liver injury (*P* < 0.01). ADAR2 KO mice displayed protection against HFD‐induced glucose intolerance, insulin resistance and dyslipidaemia. Skeletal muscle mass (*P* < 0.01), muscle strength (*P* < 0.05), muscle endurance (*P* < 0.001) and fibre size (CSA; *P* < 0.0001) were improved in ADAR2 KO mice fed with HFD compared with WT mice fed with HFD. Muscle atrophy‐associated transcripts, such as forkhead box protein O1, muscle atrophy F‐box/atrogin‐1 and muscle RING finger 1/tripartite motif‐containing 63, were decreased in ADAR2 KO mice fed with HFD compared with WT mice fed with HFD. ADAR2 deficiency attenuates HFD‐induced local liver and skeletal muscle tissue inflammation. ADAR2 deficiency abolished HFD‐induced systemic (*P* < 0.01), hepatic (*P* < 0.0001) and muscular (*P* < 0.001) SAA1 levels. C2C12 myotubes treated with recombinant SAA1 displayed a decrease in myotube length (−37%, *P* < 0.001), diameter (−20%, *P* < 0.01), number (−39%, *P* < 0.001) and fusion index (−46%, *P* < 0.01). Myogenic markers (myosin heavy chain and myogenin) were decreased in SAA1‐treated myoblast C2C12 cells.

**Conclusions:**

These results provide novel evidence that ADAR2 deficiency may be important in obesity‐associated sarcopenia and NAFLD. Increased SAA1 might be involved as a regulatory factor in developing sarcopenia in NAFLD.

## Introduction

Non‐alcoholic fatty liver disease (NAFLD) is an increasingly important cause of chronic liver disease in developed countries, affecting up to a third of the world's adult population.^1^ NAFLD is a complex multifactorial disease that often coexists with impaired glucose tolerance, dyslipidaemia, central obesity and hypertension.^2^ The hallmark of NAFLD is an excess of fatty acids in the cytoplasm of hepatocytes, which results in triglyceride (TG) accumulation (steatosis). NAFLD encompasses a broad spectrum of pathological conditions, ranging from simple steatosis (non‐alcoholic fatty liver [NAFL]) and non‐alcoholic steatohepatitis (NASH) to fibrosis/cirrhosis, which can further develop hepatocellular carcinoma and liver failure.^3^

Recent studies have reported an association between NAFLD and a reduction in muscle tissue, also known as sarcopenia.^4^ Sarcopenia is an age‐related muscle disease characterized by a decrease in muscle mass with progressive loss of muscle strength and physical performance.^5^ Besides aging, sarcopenia can occur earlier in life secondary to various causes, including an unhealthy diet, physical inactivity and metabolic disorders.^6^ The prevalence of sarcopenia is significantly increased in subjects with NAFLD and NASH compared with that in subjects without NAFLD.^7^ A diet‐induced NAFLD mouse model has demonstrated that NAFLD is associated with sarcopenia and reduced muscle strength.^8^ NAFLD and sarcopenia adversely affect metabolic health outcomes, and the coexistence of NAFLD and sarcopenia increases a two‐fold higher risk of mortality.^9^ Low skeletal muscle mass also plays a critical role in the development of NASH. Previous studies have shown a five‐fold increased risk of NAFLD in patients with sarcopenia.^10^ Although these studies demonstrated the association between NAFLD and sarcopenia, there has been insufficient evidence to support a direct causal connection between these two entities beyond their shared association with metabolic syndrome.

Adenosine deaminase acting on RNA (ADAR) catalyses a post‐transcriptional modification that converts adenosines to inosines in both coding and noncoding RNA transcripts, which is the process called adenosine‐to‐inosine (A‐to‐I) RNA editing.^11^ Three members have been identified in the human ADAR family: ADAR1 (also known as ADAR) and ADAR2 (also known as ADARB1), which are universally expressed in many tissues, and ADAR3 (also known as ADARB2), which is specifically expressed in neuronal tissue and is believed to be catalytically inactive.^12^ Abnormal ADAR expression or disrupted A‐to‐I RNA editing has been closely associated with many human diseases, including cancer, neurological disorders and metabolic disease.^13^, ^14^ ADAR1 deficiency attenuates high‐fat diet (HFD)‐induced obesity and insulin resistance (IR) in mice.^15^ A recent study done by Xiang et al. has reported that ADAR1 overexpression alleviates HFD‐induced NAFLD by inhibiting the NLRP3 inflammasome.^16^ Mice expressing either ADAR2 or inactive ADAR2 isoforms display adult‐onset obesity characterized by hyperglycaemia, hyperleptinaemia and increased adiposity.^17^ Exercise‐induced ADAR2 protects against lipogenesis during NAFLD through the editing of miR‐34a.^18^ Disruption in the expression of hepatic ADAR1 and ADAR2 could potentially lead to liver dysfunction, triggering liver inflammation and liver fibrosis.^19^

Serum amyloid A (SAA) is an acute‐phase protein with apolipoprotein properties comprising several isoforms, including SAA1, SAA2 and SAA4.^20^ SAA1 and SAA2 are mainly expressed in hepatocytes. Elevated levels of serum SAA1 have been found in patients with metabolic disorders, including obesity, IR and NAFLD.^21^, ^22^, ^23^, ^24^ Recent work by Jiang et al. has reported that knockout (KO) of SAA1/2 prevented the development of hepatic steatosis and inflammation in HFD‐induced obese mice.^25^ SAA1 is also associated with skeletal muscle wasting in cancer cachexia in mice.^26^ SAA1 interacts synergistically with interleukin‐6 (IL‐6) to contribute to angiotensin II‐induced muscle atrophy.^27^ Moreover, SAA1 mediates muscle atrophy via the Toll‐like receptor 2 (TLR2)/TLR4/nuclear factor kappa B (NF‐κB) signalling pathway in septic mice.^28^

Several studies propose that diet‐induced IR and inflammation may be the causes of concurrent skeletal muscle atrophy and liver dyslipidaemia, although there is limited evidence supporting this association. To explore whether NAFLD precedes or accompanies a dysregulation of skeletal muscle mass, strength, composition and metabolism, we utilized a previously developed and characterized mouse model of NAFLD induced by an HFD feeding. In the present study, we aimed to assess the role of ADAR2 in the development of sarcopenia in the HFD‐induced NAFLD mouse model and evaluate the functional consequences of reduced muscle mass. In addition, we assessed serum levels of SAA1, an anabolic hormone mostly synthesized in the liver, which is correlated with NAFLD and muscle atrophy.

## Materials and methods

Refer to the supporting information for detailed experimental procedures.

### Animals

ADAR2^−/−^/GluR‐B^R/R^ mice were kindly provided by Prof. Bertrand CM Tan and maintained on a B6129S genetic background. The heterozygous animals were intercrossed, producing the wild‐type (WT) (ADAR^+/+^/GluR‐B^R/R^) and homozygous mutant (ADAR2^−/−^/GluR‐B^R/R^) mice used in this study. Mice were maintained at the National Cheng Kung University Laboratory Animal Center (NCKULAC, Tainan, Taiwan), accredited by AAALAC. Mice were housed in ventilated cages (four to five mice per cage) in a temperature‐ (24 ± 1°C) and humidity‐controlled (55 ± 10%) specific‐pathogen‐free breeding unit of NCKULAC on a 12‐h light/12‐h dark cycle (light on at 7:00 AM). All animal experiments were approved by the National Cheng Kung University Institutional Animal Care and Use Committees (Approval Number: 109107) and were in accordance with local and national guidelines. Mice were randomly assigned to the normal diet (ND) and HFD feeding groups. The ND mice were fed with a standard diet (Cat. #: 5010, LabDiet, St. Louis, MO, USA), and the HFD mice were fed a diet containing 60% kcal fat (Cat. #: 58Y1, TestDiet, St. Louis, MO, USA). The experimental timelines and the details of the number of mice used in each experiment are listed in *Figure* *S1*.

### Intraperitoneal glucose tolerance test

The intraperitoneal glucose tolerance test (IPGTT) was performed as previously described.^29^, ^30^ Mice were intraperitoneally injected with a 40% sterile glucose solution dissolved in saline at a dose of 2 g/kg after 12‐h fasting. Blood samples were obtained from the tails of mice at 0, 30, 60, 90 and 120 min after glucose injection. Glucose levels were examined by using OneTouch® Ultra test strips and the OneTouch® UltraEasy blood glucose metre (LifeScan, Milpitas, CA, USA).

### Intraperitoneal insulin tolerance test

The intraperitoneal insulin tolerance test (IPITT) was performed as previously described.^29^, ^30^ Mice were intraperitoneally injected with insulin (0.75 U/kg) after 4‐h fasting. Blood samples were collected from the tails of mice at 0, 30, 60, 90 and 120 min after insulin injection. Glucose levels were measured using OneTouch® Ultra test strips and the OneTouch® UltraEasy blood glucose metre (LifeScan).

### Analysis of tissue lipid droplets

Paraffin sections (5–10 μm) of the liver, white adipose tissue (WAT) and brown adipose tissue (BAT) isolated from mice (*n* = 5) were stained with haematoxylin and eosin (H&E). The average of lipid droplets was determined by measurement of the cross‐sectional area (CSA) on ×40 bright‐field images. The analysis was done with the measurement tool within the ImageJ software.

### Grip strength test

The fore‐limb and hind‐limb grip strengths were measured with a grip strength metre (GS‐3; Bioseb, Inc., Pinellas Park, FL, USA). We performed five consecutive measurements at 1‐min intervals. In addition, grip strength was normalized for body weight.

### Rotarod test

A rotarod machine with automatic timers (RT‐01; SINGA Ltd., Taipei, Taiwan) was used. Before the training sessions, the mice were placed on a rotating cylinder (rod) for 1 min/day for two consecutive days. For the training sessions, mice were acclimatized for 3 min/day on the rotarod instrument set to rotate starting from 1 to 15 rpm (the speed of 1 rpm every 2 s) for three consecutive days. In the five following days, mice were tested with accelerating speed (1–40 rpm in 3 min with directional reversal). Mice were tested three times in a row, with 10 min of rest between each trial. The time the mice spent on the rod before falling was recorded and averaged over the 5 days.

### Muscle histology

The gastrocnemius (GA) muscle was dissected from the mice, weighted, slowly frozen in isopentane, chilled in liquid nitrogen, embedded in optimal cutting temperature (OCT) and stored at −80°C. The 5‐μm‐thick transverse muscle sections were sliced with a cryostat microtome and stained with H&E for the analysis of myofiber CSA.

For myofiber CSA frequency distribution analysis, the 5‐μm‐thick transverse muscle sections were incubated overnight at 4°C with a primary antibody against laminin. Slides were then washed three times in 5 min in phosphate‐buffered saline (PBS) and incubated for 1 h in the dark with goat anti‐rabbit IgG Alexa Fluor 594. Images were obtained using an Olympus upright fluorescent microscope (Olympus Corporation, Tokyo, Japan). Myofiber CSA was analysed using ImageJ software (NIH, Bethesda, MA, USA).

For myosin heavy chain (MyHC) fibre type analysis, the transverse muscle sections (5 μm) were incubated for 2 h at room temperature with primary antibodies for MHC‐I (1:200, Cat. #: BA‐F8, DSHB) and MHC‐II (1:200, Cat. #: SC‐71, DSHB). Slides were then washed three times for 5 min in PBS and incubated for 1 h in the dark with chicken anti‐mouse IgG Alexa Fluor 647 and goat anti‐mouse Alexa Fluor 488 diluted in CAS‐Block™ Histochemical Reagent (Cat. #:008120, Invitrogen). Nuclei were stained using Hoechst 33258. Images were obtained using an Olympus upright fluorescent microscope (Olympus Corporation). Fibre type percentages were analysed using ImageJ software (NIH).

### Statistical analysis

Statistical analyses were performed with GraphPad Prism 8.0 (GraphPad Software, San Diego, CA, USA). The total number (*N*) of observations in each group was indicated in the figures. The significance was set at *P* < 0.05. Student's *t*‐test was adopted to analyse the data sets with a single factor (the HFD effect). The body weight of mice was analysed using a repeated measured two‐way analysis of variance (ANOVA). Sidak's post hoc test was used to perform multiple comparison analysis after the two‐way ANOVAs.

## Results

### ADAR2 knockout reduces high‐fat diet‐induced weight gain in male mice

**Figure 1:**
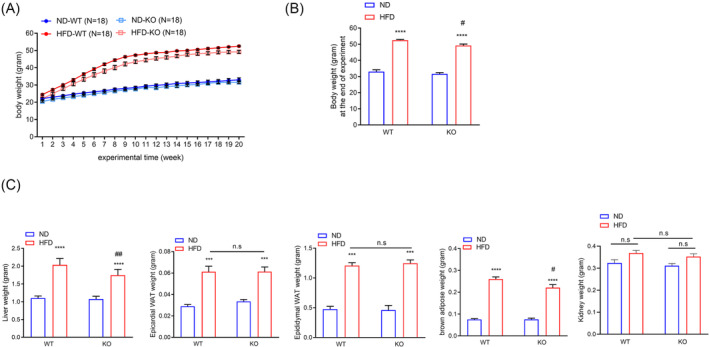
ADAR2 KO decreased body weight and liver weight in male obese mice. Physiological parameters in mice from the age of 5–25 weeks. (A) Body weight of mice during the feedings. *n* = 18 mice per group. (B) Quantitative results of the body weight of mice after the end of the regimen. *n* = 18 mice per group. (C) Weights of liver, epididymal adipose, epicardial adipose, BAT and kidney derived from WT and ADAR2 KO mice fed with ND or HFD are shown. *n* = 18 mice per group. All data are expressed as the mean ± SEM. Tukey's multiple comparison test after the two‐way ANOVA was conducted for (A)–(C). *ND‐WT group versus HFD‐WT group or ND‐KO group versus HFD‐KO group; ***P < 0.001, ****P < 0.0001; ^#^HFD‐WT group versus HFD‐KO group; ^#^ *P* < 0.05, ^##^ *P* < 0.01; n.s., not significant.

To evaluate the possible physiological function of ADAR2 in the association between NAFLD and sarcopenia, we used an HFD‐induced NAFLD mouse model. This model is a widely used animal model of NAFLD, showing obesity, IR and hepatic inflammation after 16 weeks of feeding.^31^ We first investigated the body weight, water intake, food intake and energy intake of mice fed with either an ND or HFD from the age of 5 weeks until 25 weeks. As expected, both male and female mice gained significantly more body weight when fed an HFD at 20 weeks, and the body weight of ADAR^−/−^/GluR‐B^R/R^ (ADAR2 KO) mice was lower than that of ADAR^+/+^/GluR‐B^R/R^ (WT) mice in male mice but not in female mice after HFD feeding (*Figure* *1* *A,B*, male; *Figure* *S2A,B*, female). Daily food and water intake showed no significant differences between each group in both male and female mice (*Figure* *S3A–C*, male; *Figure* *S3D–F*, female).

### ADAR2 knockout decreases liver weight in male obese mice but not in female obese mice

Next, we characterized whether ADAR2 KO impacted the organ weight under HFD. The liver weight of male ADAR2 KO mice fed with HFD was significantly lower than that of male WT mice fed with HFD (*Figure* *1* *C*). The liver weight showed no significant differences between WT mice fed with HFD and ADAR2 KO mice fed with HFD in female mice (*Figure* *S2B*). Both epicardial WAT and epididymal WAT and kidney were not significantly different between ADAR2 KO‐HFD mice and WT‐HFD mice in both sexes (*Figure* *1* *C*, male; *Figure* *S2B*, female). The BAT weight of male ADAR2 KO mice fed with HFD was significantly reduced compared with that of male WT mice fed with HFD (*Figure* *1* *C*). The BAT weight showed no significant differences between WT mice fed with HFD and ADAR2 KO mice fed with HFD in female mice (*Figure* *S2C*).

### ADAR2 knockout ameliorates high‐fat diet‐induced glucose/lipid‐metabolic dysfunctions in male obese mice

**Figure 2:**
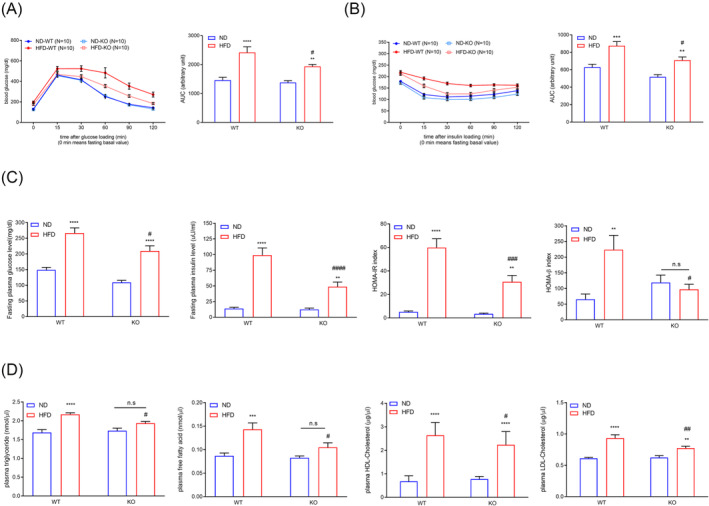
ADAR2 KO ameliorated blood glucose levels, blood insulin levels and serum lipid levels in male obese mice. (A) Blood glucose levels during IPGTT in male mice (left panel). Analysis of the area under the curve (AUC) of IPGTT results (right panel). *n* = 10 mice per group. (B) Blood glucose levels during IPITT in male mice (left panel). Analysis of the AUC of IPGTT results (right panel). *n* = 10 mice per group. (C) Fasting plasma glucose levels, fasting plasma insulin levels, calculated HOMA‐IR index and calculated HOMA‐β index of male mice. *n* = 10 mice per group. (D) Plasma levels of TG, free fatty acid, HDL‐cholesterol and LDL‐cholesterol in mice. *n* = 10 mice per group. All data are expressed as the mean ± SEM. Tukey's multiple comparison test after the two‐way ANOVA was conducted for (A)–(D). *ND‐WT group versus HFD‐WT group or ND‐KO group versus HFD‐KO group; **P < 0.01, ***P < 0.001, ****P < 0.0001; ^#^HFD‐WT group versus HFD‐KO group; ^#^ *P* < 0.05, ^##^ *P* < 0.01, ^###^ *P* < 0.001, ^####^ *P* < 0.0001; n.s., not significant.

As obesity impacts glucose metabolism and insulin action, we next analysed the capacity of the mice to break down glucose by performing a glucose tolerance test (GTT) on mice fed with ND or HFD for 20 weeks. After 20 weeks of HFD feeding, male ADAR2 KO mice displayed improvements in glucose tolerance (*Figure* *2* *A*) when compared with WT mice, but both female WT and female ADAR2 showed a similar pattern of glucose intolerance after 20 weeks of HFD feeding (*Figure* *S4A*). We then tested the insulin sensitivity of ADAR2 KO mice by performing an insulin tolerance test (ITT). We found that male ADAR2 KO mice increased insulin sensitivity under HFD (*Figure* *2* *B*). In contrast, ADAR2 KO did not enhance insulin sensitivity in female mice fed with HFD (*Figure* *S4B*). Moreover, HFD led to upregulated fasting plasma glucose levels in WT mice and ADAR2 KO mice compared with the ND control, but ADAR2 KO did not lower fasting plasma glucose levels in female WT mice fed with HFD (*Figure* *S4C*). Hence, we focused on males for the rest of the studies. Our plasma biochemical examinations showed elevated levels of plasma glucose, insulin (*Figure* *2* *C*), total cholesterol, free fatty acid and TGs in the HFD obese mice (*Figure* *2* *D*). ADAR2 KO attenuated these metabolic dysregulations (*Figure* *2* *C,D*). Furthermore, the homeostasis model assessment‐estimated insulin resistance (HOMA‐IR) index was calculated to evaluate systemic IR. Compared with the ND group, the HOMA‐IR value was significantly increased with HFD feeding, whereas the HOMA‐IR value had a lower induction upon the HFD diet in ADAR2 KO mice (*Figure* *2* *C*). The HOMA‐β index was calculated to evaluate beta‐cell function. Compared with the ND group, the HOMA‐β value was significantly increased with HFD feeding, whereas ADAR2 silencing completely abolished the HFD‐induced effect in ADAR2 KO mice (*Figure* *2* *C*).

### ADAR2 knockout attenuates high‐fat diet‐induced hepatic lipid accumulation and injury in mice

**Figure 3:**
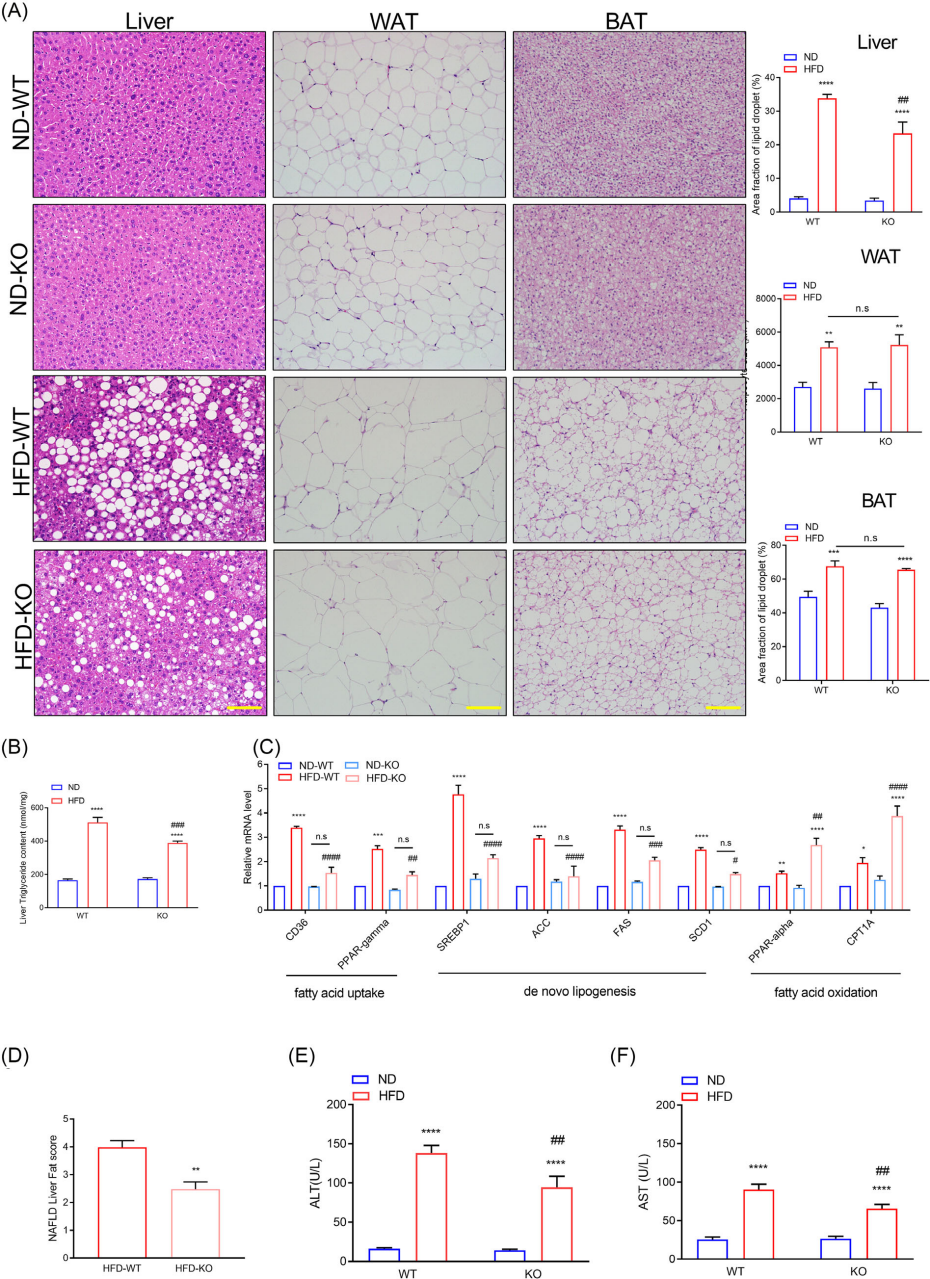
ADAR2 KO alleviated hepatic steatosis and injury in mice fed with HFD. (A) Representative H&E staining of liver, WAT and BAT derived from WT and ADAR2 KO mice fed with ND or HFD is shown (left panel) and quantitative analysis of WAT size of different groups is shown (right panel). Scale bars = 100 μm. *n* = 5 mice per group. (B) Quantitative results of the liver TG content in each group. *n* = 5 mice per group. (C) Quantitative PCR was performed to determine the hepatic mRNA levels of genes related to fatty acid metabolism (CD36, PPAR‐gamma, SREBP1, ACC, FAS, SCD1, PPAR‐alpha and CPT1A) in mice from the indicated groups. Gene expression was normalized to GAPDH mRNA levels. *n* = 6 mice per group. (D) NAFLD activity score. *n* = 5 mice per group. (E, F) Serum levels of AST and ALT were measured in mice after 20 weeks of ND diet feeding or HFD challenge. *n* = 9 mice per group. All data are expressed as the mean ± SEM. Tukey's multiple comparison test after the two‐way ANOVA was conducted for (A)–(C), (E) and (F). Unpaired two‐tailed Student's *t*‐test was conducted for (D). *ND‐WT group versus HFD‐WT group or ND‐KO group versus HFD‐KO group; *P < 0.05, **P < 0.01, ***P < 0.001, ****P < 0.0001; ^#^HFD‐WT group versus HFD‐KO group; ^#^ *P* < 0.05, ^##^ *P* < 0.01, ^###^ *P* < 0.001, ^####^ *P* < 0.0001; n.s., not significant.

As shown in *Figure* *1* *C*, male ADAR2 KO mice fed with HFD had a lower liver weight when compared with male WT mice fed with HFD. Histological analyses further showed that lipid deposition was also substantially reduced in the liver of ADAR2 KO mice fed with HFD compared with that of WT mice fed with HFD, but not in WAT and BAT (*Figure* *3* *A*). Hepatic TG content was reduced in ADAR2 KO mice fed with HFD compared with that of WT mice fed with HFD (*Figure* *3* *B*). Quantitative PCR (qPCR) assays showed that hepatic mRNA levels of genes related to fatty acid uptake and synthesis (CD36, peroxisome proliferator‐activated receptor gamma [PPAR‐gamma], sterol regulatory element binding protein 1 [SREBP1], acetyl‐CoA carboxylase [ACC], fatty acid synthase [FAS] and stearoyl CoA desaturase 1 [SCD1]) were significantly diminished in the liver of ADAR2 KO mice fed with HFD compared with that of WT mice fed with HFD. Additionally, the mRNA levels of genes related to lipid β‐oxidation (PPAR‐alpha and carnitine palmitoyltransferase 1a [CPT1A]) were increased in the liver of ADAR2 KO mice fed with HFD compared with that of WT mice fed with HFD (*Figure* *3* *C*). Furthermore, a reduction in NAFLD score in ADAR2 KO mice fed with HFD was observed in comparison with the WT mice fed with HFD (*Figure* *3* *D*). The serum levels of alanine aminotransferase (ALT) and aspartate aminotransferase (AST) were reduced in ADAR2 KO mice fed with HFD compared with WT mice fed with HFD (*Figure* *3* *E,F*). These results suggested that ADAR2 KO attenuated hepatic steatosis and injury induced by metabolic stress in mice.

### ADAR2 knockout recovers physical performance and skeletal muscle mass loss in high‐fat diet‐induced non‐alcoholic fatty liver disease mice

**Figure 4:**
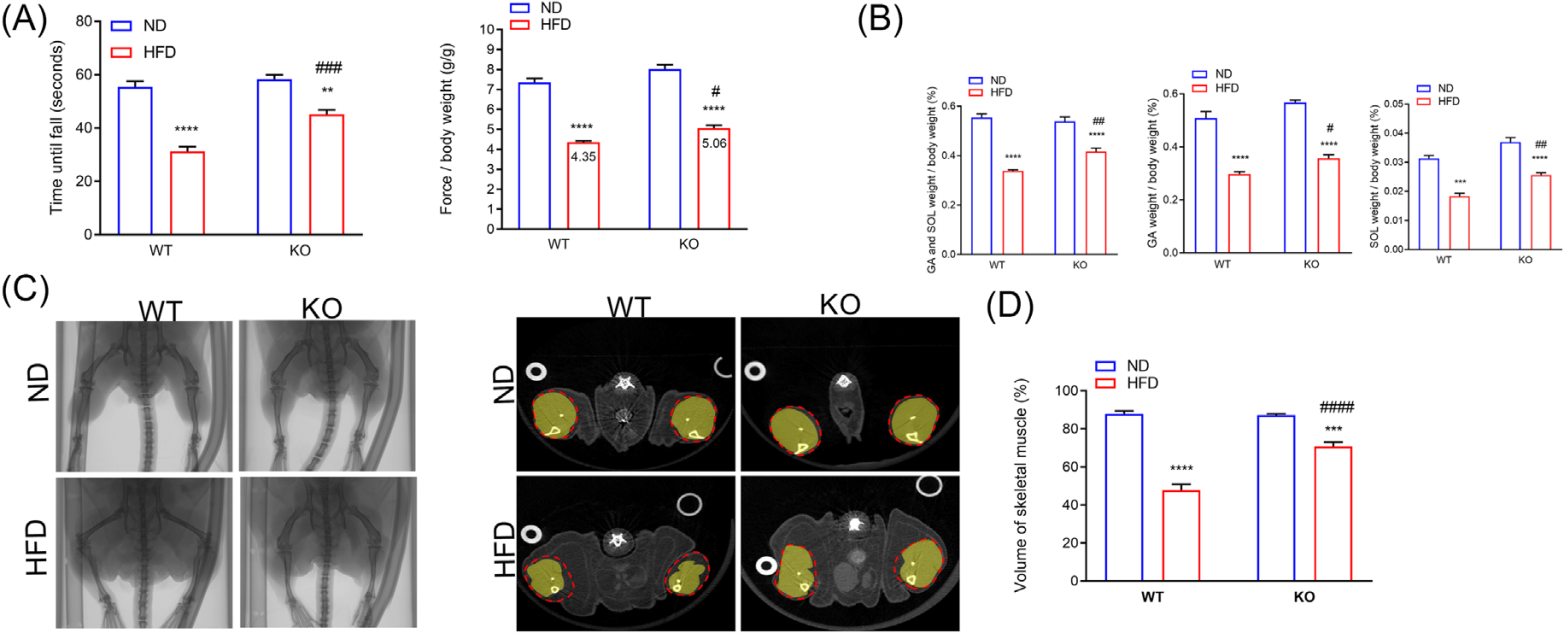
ADAR2 KO increased muscle endurance, muscle strength and skeletal muscle mass. (A) Rotarod performance (right panel) and fore‐limb grip strength (left panel). *n* = 10 mice per group. (B) Relative gastrocnemius and soleus muscle weight in each group. *n* = 10 mice per group. (C) Scanned raw images were reconstructed in 3D and analysed using MicroView analysis software (left panel). The cross sections of the calf were represented (middle panel). The whole calf was circled with a dotted line, and the skeletal muscle was marked yellow. *n* = 6 mice per group. (D) Percentage of skeletal muscle volume in the whole calf (right panel). *n* = 6 mice per group. All data are expressed as the mean ± SEM. Tukey's multiple comparison test after the two‐way ANOVA was conducted for (A)–(D). *ND‐WT group versus HFD‐WT group or ND‐KO group versus HFD‐KO group; **P < 0.01, ***P < 0.001, ****P < 0.0001; ^#^HFD‐WT group versus HFD‐KO group; ^#^ *P* < 0.05, ^##^ *P* < 0.01, ^###^ *P* < 0.001, ^####^ *P* < 0.0001.

To determine whether silencing of ADAR2 modulates the physical performance in HFD‐induced NAFLD mice, we compared fore‐limb grip strength and rotarod performance. The ADAR2 KO mice fed with HFD displayed a significant increase in fore‐limb grip strengths compared with WT mice fed with HFD (*Figure* *4* *A*). Additionally, ADAR2 KO mice fed with HFD performed better in rotarod tests than WT mice fed with HFD (*Figure* *4* *A*). The GA and soleus muscle weights of both WT mice and ADAR2 KO mice fed with HFD were lower than those of both WT mice and ADAR2 KO mice fed with ND. Furthermore, the GA and soleus muscle weights of ADAR2 KO mice fed with HFD were higher than those of WT mice fed with HFD (*Figure* *4* *B*). We explored the possibility of evaluating the hind‐limb muscle mass through non‐invasive microcomputed tomography (micro‐CT) technology (*Figure* *4* *C*). Our results showed that a decrease in the volume of hind‐limb muscle was observed in both WT mice and ADAR2 KO mice fed with HFD, which was less pronounced when ADAR2 was silenced (*Figure* *4* *C,D*).

### ADAR2 knockout recovers high‐fat diet‐induced loss of muscle cross‐sectional area

**Figure 5:**
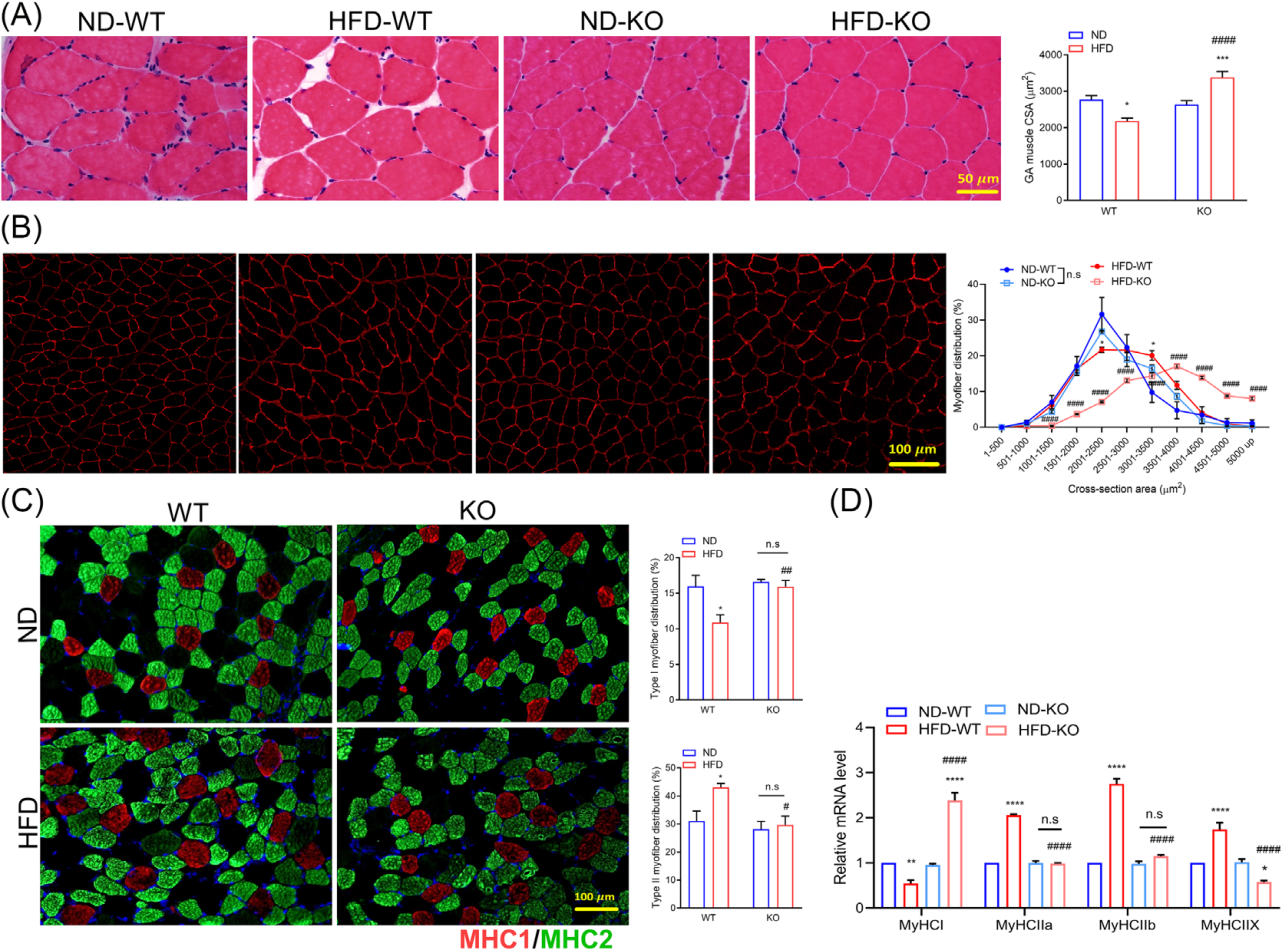
ADAR2 KO prevented HFD‐induced myofiber atrophy. (A) Representative cross sections and CSA quantification from the GA muscle of mice. Scale bar = 50 μm. *n* = 6 mice per group. (B) Representative images of cross sections and myofiber distribution frequency from the GA muscle of mice (red = laminin). Scale bar = 100 μm. *n* = 5 mice per group. (C) Representative images of fibre type staining of GA muscles (red = type 1 fibres; green = type 2A fibres; blue = cell nuclei). Scale bars = 100 μm (left panel). Representative quantification of MyHC fibre type from the GA muscles of mice (right panel). *n* = 5 mice per group. (D) Quantitative RT‐PCR analysis of MyHCI and MyHCIIa/b/x, respectively. *n* = 6 mice per group. All data are expressed as the mean ± SEM. Tukey's multiple comparison test after the two‐way ANOVA was conducted for (A), (C) and (D). *ND‐WT group versus HFD‐WT group or ND‐KO group versus HFD‐KO group; *P < 0.05, **P < 0.01, ***P < 0.001, ****P < 0.0001; ^#^HFD‐WT group versus HFD‐KO group; ^#^ *P* < 0.05, ^##^ *P* < 0.01, ^####^ *P* < 0.0001. Tukey's multiple comparison test after the two‐way ANOVA was conducted for (B). ^$^ND‐WT group versus HFD‐WT group, ^$^ *P* < 0.05; *ND‐KO group versus HFD‐KO group, **P < 0.01, ****P < 0.0001; ^#^HFD‐WT group versus HFD‐KO group; ^####^ *P* < 0.0001; n.s., not significant.

We investigated skeletal muscle fibre CSA in WT and ADAR2 KO mice fed with HFD as well as muscle fibre type (MyHC). Similar to changes in muscle weight, CSA was decreased in WT mice fed with HFD compared with WT mice fed with ND. Not only was CSA completely preserved in ADAR2 KO mice fed with HFD compared with ADAR2 KO mice fed with ND, but it was also increased (*Figure* *5* *A*). We also performed a myofiber CSA frequency distribution analysis and found a rightward shift in myofiber CSA from ADAR2 KO mice fed with HFD compared with WT mice fed with HFD, suggesting that ADAR2 KO led to larger fibres under HFD feeding (*Figure* *5* *B*). In WT mice, the HFD resulted in a decrease in type 1 fibres and an increase in type 2 fibres. In contrast, ADAR2 KO mice did not exhibit these changes when fed with HFD (*Figure* *5* *C*). Similarly, qPCR results showed that in WT mice, the HFD led to a decrease in MyHC1 and an increase in MHC2a, 2b and 2x. The pattern is different in ADAR2 KO mice, with a substantial increase in MyHC1 and a decrease in MyHC2a, 2b and 2x (*Figure* *5* *D*). We also found that ADAR2 KO upregulated some genes associated with slow‐twitch fibres and downregulated some genes associated with fast‐twitch fibres (*Figure* *S5*). These results showed that ADAR2 KO affected the composition of skeletal muscle fibre types and promoted the expression of fast‐twitch fibres in vivo.

### ADAR2 knockout improves high‐fat diet‐induced muscle atrophy through the AKT/forkhead box protein O1 pathway

**Figure 6:**
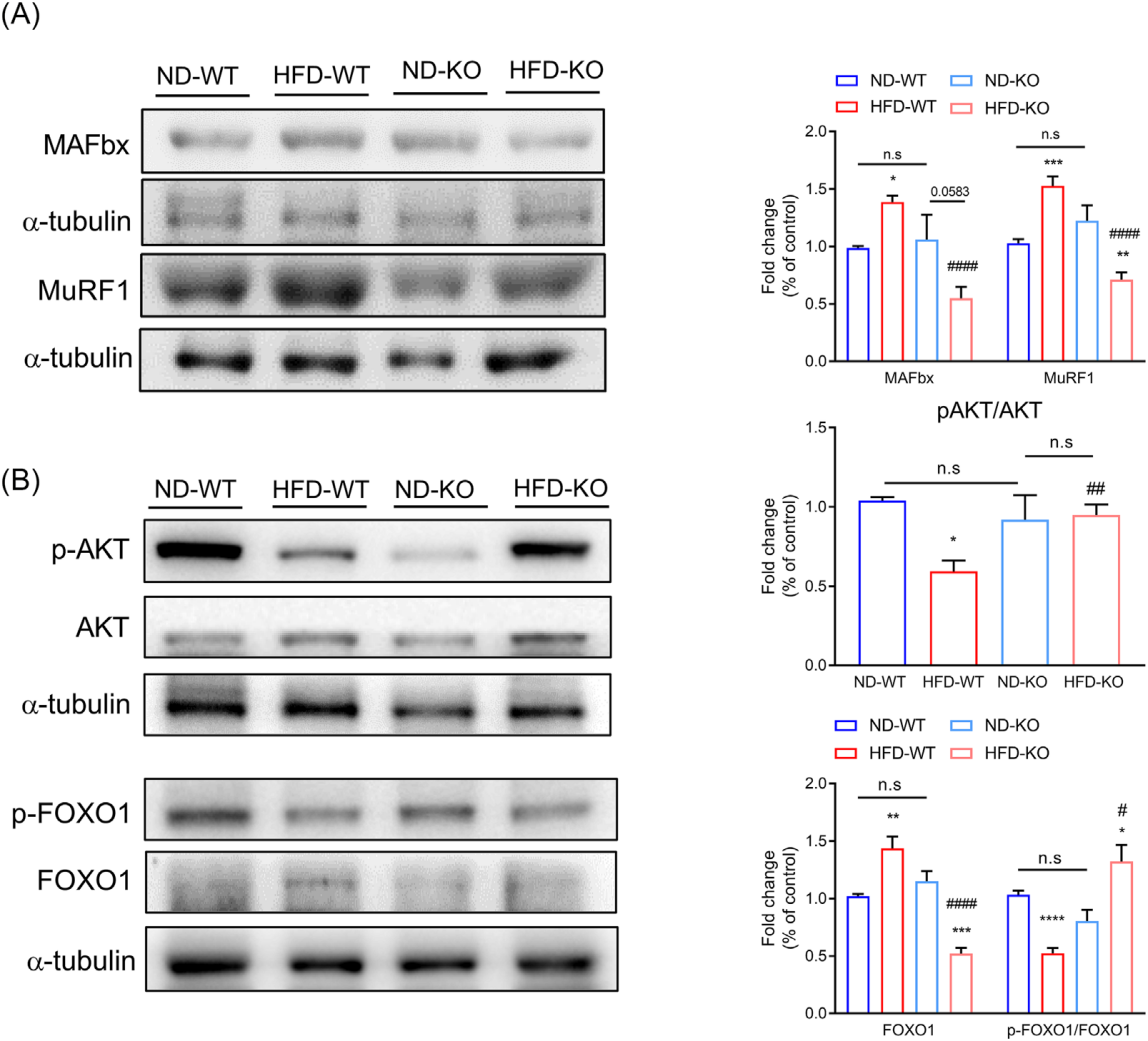
ADAR2 KO improved HFD‐induced muscle atrophy through the AKT/FOXO1 pathway. (A) Western blot analysis of the protein expression of muscle atrophy markers (MAFbx and MuRF1) using tissue lysates of GA muscle in each group (*n* = 6 mice per group). (B) Western blot analysis of the protein expression p‐FOXO, FOXO1, p‐Akt and Akt using tissue lysates of GA muscle in each group. α‐Tubulin was used as a loading control (*n* = 6 mice per group). All data are presented as the mean ± SEM. Unpaired two‐tailed Student's *t*‐test was conducted for (A) and (B). *ND‐WT group versus HFD‐WT group or ND‐KO group versus HFD‐KO group; *P < 0.05, **P < 0.01, ***P < 0.001, ****P < 0.0001; ^#^HFD‐WT group versus HFD‐KO group; ^#^ *P* < 0.05, ^##^ *P* < 0.01, ^#####^ *P* < 0.0001; n.s., not significant.

Muscle mass is determined by a balance between protein synthesis (anabolism) and protein degradation (catabolism). We next examined the protein level of muscle atrophy markers, including muscle atrophy F‐box (MAFbx)/atrogin‐1 and muscle RING finger 1 (MuRF1) in GA muscle, because they are muscle‐specific E3 ligases responsible for protein degradation. As expected, expression of atrogin‐1/MAFbx and MuRF1 was increased in the GA muscle of WT mice fed with HFD but abolished in the GA muscle of ADAR2 KO mice fed with HFD (*Figure* *6* *A*). To further investigate the mechanism through which ADAR2 ameliorated HFD‐induced muscle atrophy, proteins in the AKT/forkhead box protein O1 (FOXO1) signalling pathways were evaluated. The expression levels of p‐AKT were reduced in the HFD‐WT group, whereas ADAR2 KO significantly increased the expression levels of p‐AKT (*Figure* *6* *B*). We also found that HFD not only decreased the levels of p‐FOXO1 but also upregulated FOXO1 in GA muscle, whereas ADAR2 KO reversed these effects (*Figure* *6* *B*). Our results indicated that ADAR2 KO ameliorates protein degradation through the AKT/FOXO1 pathway.

### ADAR2 knockout mitigates a high‐fat diet‐induced inflammatory response

**Figure 7:**
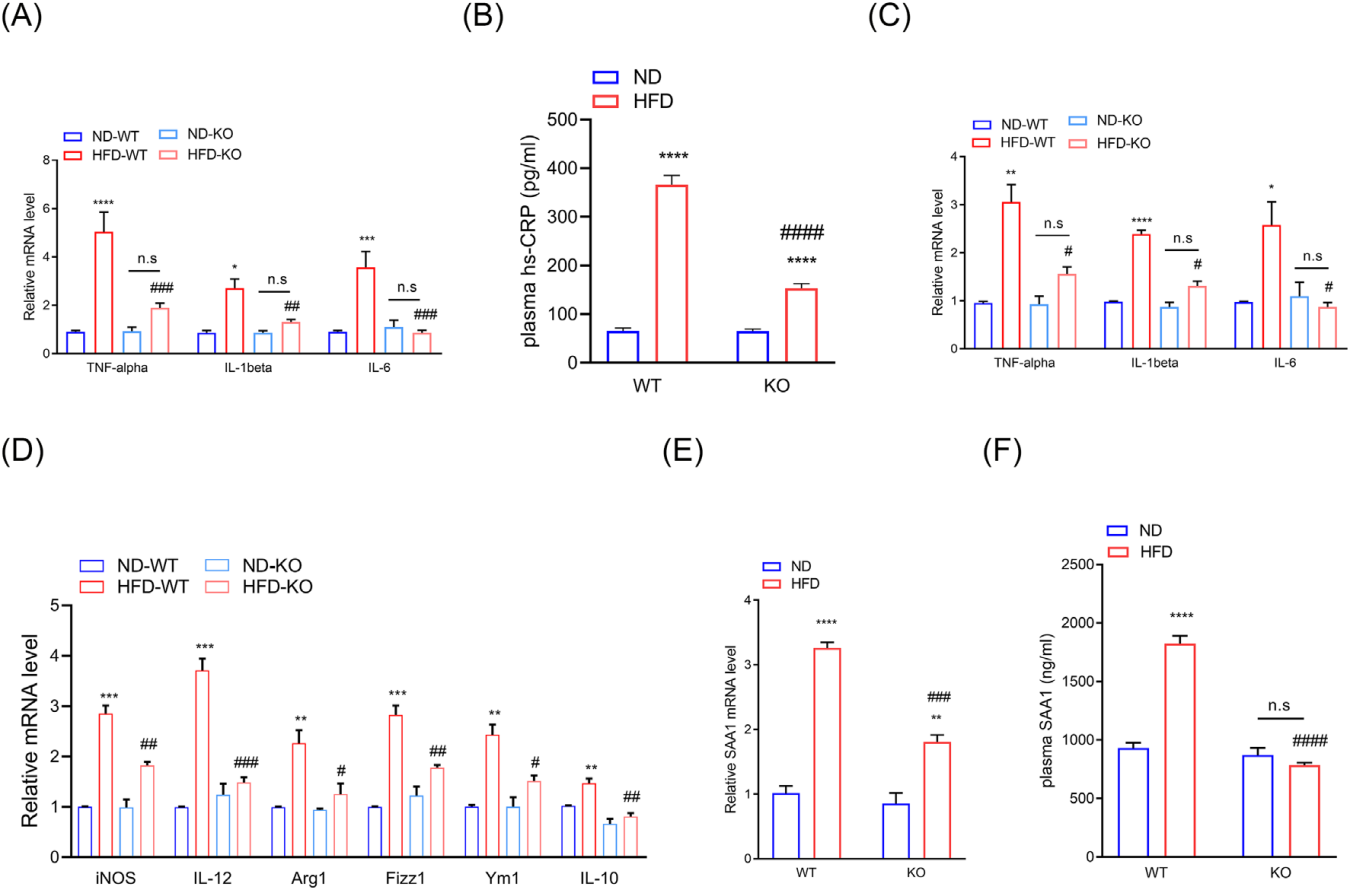
ADAR2 KO mitigated the HFD‐induced inflammatory response. (A) Quantitative PCR was performed to determine the hepatic mRNA levels of genes related to inflammation (TNF‐α, IL‐1β and IL‐6) in mice from the indicated groups. Gene expression was normalized to GAPDH mRNA levels. *n* = 6 mice per group. (B) Plasma levels of CRP in mice from the indicated groups. *n* = 6 mice per group. (C) Quantitative PCR was performed to determine the mRNA levels of genes related to inflammation (TNF‐α, IL‐1β and IL‐6) in the GA muscle of mice from the indicated groups. Gene expression was normalized to GAPDH mRNA levels. *n* = 6 mice per group. (D) Quantitative PCR was performed to determine the mRNA levels of genes related to macrophages (M1: iNOS and IL‐12; M2: Arg‐1, Fizz1, Ym1 and IL‐10) in the GA muscle of mice from the indicated groups. Gene expression was normalized to GAPDH mRNA levels. *n* = 6 mice per group. (E) Plasma concentrations of SAA1 from ADAR2 KO and control mice treated with ND or HFD. *n* = 10 mice per group. (F) Quantitative PCR was performed to determine the hepatic mRNA levels of SAA1 in mice from the indicated groups. *n* = 6 mice per group. Each value represents the mean ± SEM from at least three independent experiments. Tukey's multiple comparison test after the two‐way ANOVA was conducted for (A)–(F). *ND‐WT group versus HFD‐WT group or ND‐KO group versus HFD‐KO group; *P < 0.05, **P < 0.01, ***P < 0.001, ****P < 0.0001; ^#^HFD‐WT group versus HFD‐KO group; ^#^ *P* < 0.05, ^##^ *P* < 0.01, ^###^ *P* < 0.001, ^####^ *P* < 0.0001; n.s., not significant.

Chronic inflammation could serve as an important association between sarcopenia and NASH.^32^ We further investigated the role of ADAR2 in obesity‐induced chronic inflammation. Our results showed that the HFD‐induced accumulation of mRNA levels encoding tumour necrosis factor‐alpha (TNF‐α), IL‐1β and IL‐6 was abolished when ADAR2 was silenced (*Figure* *7* *A*). As shown in *Figure* *7* *B*, HFD‐induced upregulation of plasma high‐sensitivity C‐reactive protein (hs‐CRP) levels was abolished when ADAR2 was silenced. Similar results were observed in GA muscle, showing that ADAR2 KO reduced the HFD‐induced accumulation of mRNA levels encoding TNF‐α, IL‐1β and IL‐6 (*Figure* *7* *C*). Therefore, the gene expression of macrophage‐specific markers, including inducible nitric oxide synthase (iNOS), IL‐12, Arg‐1, Fizz1, Ym1 and IL‐10, was determined in GA muscle by real‐time qPCR (*Figure* *7* *D*). Our results demonstrated that the expression of macrophage M1 markers (iNOS and IL‐12) was increased in WT mice fed with HFD; however, the ADAR2 KO decreased their expression (*Figure* *7* *D*). At the same time, the levels of macrophage M2 markers (Arg‐1, Fizz1, Ym1 and IL‐10) were downregulated by ADAR2 KO compared with the HFD group (*Figure* *7* *D*). Furthermore, ADAR2 KO significantly diminishes HFD‐induced SAA1 levels in both the liver and plasma (*Figure* *7* *E,F*). These data suggest that inflammation can be involved in the pathogenesis of NAFLD and sarcopenia.

### Serum amyloid A1 potentially contributes to non‐alcoholic fatty liver disease‐associated sarcopenia

**Figure 8:**
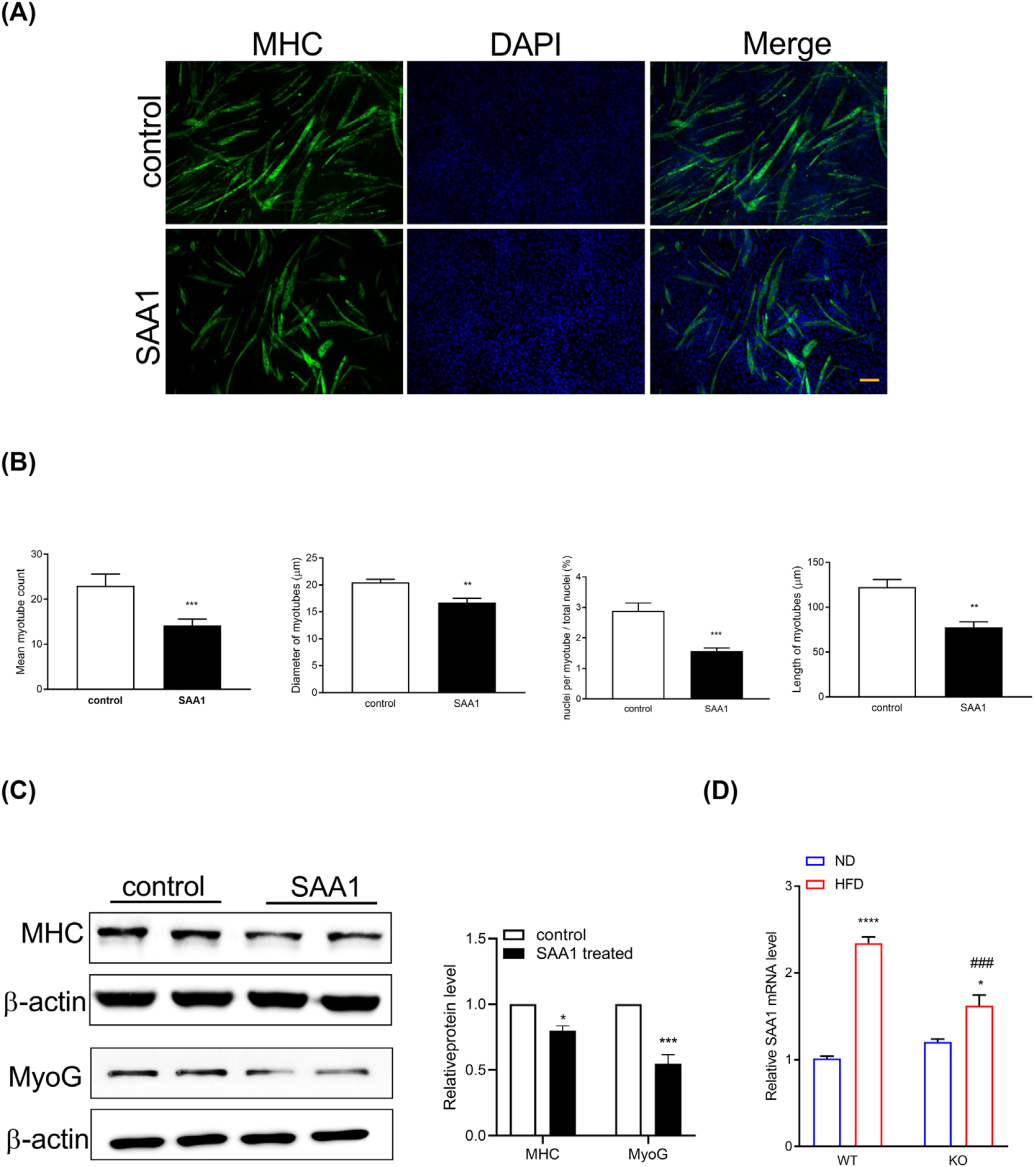
Recombinant SAA1 treatment of C2C12 myoblasts resulted in smaller myotubes. C2C12 cells were differentiated for 5 days and treated with 10 μg/mL recombinant SAA1 or vehicle (control) for 72 h. (A) Representative images of immunofluorescence staining with an anti‐myosin heavy chain (MyHC) antibody (green). Nuclei were stained with DAPI (blue). Scale bar = 100 μm. (B) Representative quantifications of diameter, length, number and fusion index were measured. (C) Western blot analysis of the protein expression of myogenic markers (MHC and MyoG) in lysates from C2C12 cells. (D) Quantitative PCR was performed to determine the mRNA levels of SAA1 in the GA muscle of mice from the indicated groups. Each value represents the mean ± SEM from at least three independent experiments. Unpaired two‐tailed Student's *t*‐test was conducted for (B) and (C). *Control group versus SAA1 group; *P < 0.05, **P < 0.01, ***P < 0.001; Tukey's multiple comparison test after the two‐way ANOVA was conducted for (D). *ND‐WT group versus HFD‐WT group or ND‐KO group versus HFD‐KO group; *P < 0.05, ****P < 0.0001; ^#^HFD‐WT group versus HFD‐KO group; ^###^ *P* < 0.001.

Skeletal muscle atrophy is caused by inflammatory cytokines such as IL‐1β and IL‐6.^27^ SAA1 is associated with muscle wasting in cancer cachexia and sepsis.^26^, ^33^ We hypothesized that upregulated SAA1 might be a regulator leading to muscle atrophy in obese‐induced NAFLD. To test the hypothesis that SAA1 mediates its atrophic effects on myocytes, we treated C2C12 myotubes with recombinant mouse SAA1. The effect of SAA1 in skeletal myogenesis was evaluated by immunolabelling differentiated myotubes with an anti‐MyHC antibody (*Figure* *8* *A*). Myotubes treated with 100 nM of SAA1 displayed reduced number, diameter, length and fusion index compared with the control (*Figure* *8* *B*). We also observed the downregulation of myogenic markers (MHC and myogenin [MyoG]) in SAA1‐treated myoblast C2C12 cells (*Figure* *8* *C*). Our results showed that SAA1 treatment caused C2C12 myotube atrophy, which concurs with a previous study.^28^ A similar result was obtained in isolated mouse myoblasts treated with SAA1 (*Figure* *S6*). Furthermore, we quantitated the expression of SAA1 in the GA muscle of each group by qPCR. Our results showed that the HFD‐induced expression of SAA1 was reduced when ADAR2 was silenced (*Figure* *8* *D*).

## Discussion

NAFLD, characterized by excessive fat accumulation in the liver, is now the most common cause of chronic liver disease worldwide. Increased body weight, metabolic syndrome and older age are the most common and strongest risk factors for NAFLD. Sarcopenia, the age‐related decline in muscle mass and strength, has been found to be a risk factor for NAFLD since the early stages of the disease. Sarcopenia and NAFLD are growing health problems and share common underlying mechanisms. However, there is not sufficient data to support a direct causal relationship between sarcopenia and NAFLD. In the present study, we found that ADAR2 deficiency alleviated obesity‐associated sarcopenia and NAFLD. Genetic ADAR2 KO obese mice showed improved metabolic disorders, including hyperglycaemia, hyperlipidaemia, IR, hepatic lipid accumulation and injury. Furthermore, KO of ADAR2 alleviated HFD‐induced skeletal muscle atrophy, muscle strength and muscle endurance. KO of ADAR2 reduced systemic SAA1 and hepatic SAA1 levels in obese mice. Our results showed that SAA1 caused the atrophy of C2C12 myotubes. Importantly, we determined that increased SAA1 might be involved as a factor in developing sarcopenia in NAFLD.

Chronic inflammatory responses are shared, and mutually perpetuating pathogenetic mechanisms are involved in IR, sarcopenia and NAFLD. Studies have demonstrated the association of high systemic levels of cytokines, including TNF‐α, hs‐CRP and IL‐6, with low muscle mass and a progressive course of NAFLD.^34^ Previous studies have shown that inflammatory factors (IL‐1β, IL‐6 and TNF‐α) can induce the expression of hepatic SAA1.^35^ SAA1 has been reported to be involved in type 2 diabetes and NAFLD. Jiang et al. demonstrated that SAA1 was increased in the NAFLD liver in both humans and mice, and SAA1/1 deficiency alleviated HFD‐induced metabolic disorder, hepatic steatosis and inflammation. In the present study, we found that a high level of SAA1 was expressed in the plasma and fatty liver tissue of obese‐induced NAFLD mice. ADAR2 KO abolished HFD‐induced upregulated levels of SAA1. It is shown that SAA1 induces muscle atrophy in vitro, which is mediated by TLR2 and TLR4 as well as NF‐κB.^28^ Consistent with this finding, our results also showed that recombinant SAA1 caused C2C12 myotube atrophy. In HFD‐induced NAFLD mice, we found high circulating levels of SAA1, which is mainly produced by the liver, indicating the contributions of other organs to muscle wasting. Additional studies will be needed to determine the underlying mechanisms by which ADAR2 KO affects the production of SAA1 in HFD‐induced NAFLD mice.

Macrophages play essential roles in the activation and protection of muscle fibres after muscle inflammation and injury.^36^ There are two phenotypes of activated macrophages, namely, proinflammatory M1 (classical activation) and anti‐inflammatory M2 (alternative activation).^37^ M1 macrophages mostly secrete proinflammatory cytokines, including TNF‐α, IL‐1β and IL‐6, consequently resulting in acceleration of myofiber lysis and protein degradation. M2 macrophages can release anti‐inflammatory cytokines, such as transforming growth factor‐beta (TGF‐β), IL‐10 and insulin‐like growth factor‐1 (IGF‐1), contributing to myogenesis and tissue repair. In chronic muscle damage, both M1 and M2 polarized macrophages coexist in the tissue but fail to promote tissue repair and homeostasis recovery. In this study, iNOS, IL‐12, Arg‐1, Fizz1, Ym1 and IL‐10 were detected as markers of M1 macrophages and M2 macrophages, respectively. We observed the increase of M1 macrophages and M2 macrophages in the GA muscle of WT mice fed with HFD. ADAR2 KO abolished HFD‐induced upregulated levels of M1 macrophages and M2 macrophages. Therefore, a definitive understanding of the complex temporally coordinated macrophage roles in NAFLD‐related sarcopenia and the balance of M1 and M2 macrophages is crucial to muscle recovery.

Mammalian skeletal muscle is typically composed of different types of muscle fibre, which display different metabolic and contractile characteristics. Slow‐twitch (type I) muscle fibres are rich in mitochondria and display high oxidative capacity and strong anti‐fatigue ability, whereas fast‐twitch (type II) muscle fibres have lower mitochondrial and capillary density and generate ATP primarily through glycolysis.^38^ Indeed, fibre type distribution has been reported to directly correlate with glucose uptake and IR in humans.^39^ Studies have reported that individuals with type 2 diabetes mellitus or obesity have more glycolytic fibres and fewer oxidative fibres than healthy individuals. A previous study revealed that subjects with metabolic syndrome had lower type I fibre content and higher type IIa fibre content than control subjects.^40^ In the present study, WT mice fed with HFD had reduced slow type I skeletal muscle fibres and increased type II muscle fibres, whereas ADAR2 KO mice fed with HFD had increased type I skeletal muscle fibres and decreased type II skeletal muscle fibres. Skeletal muscle fibre type can affect the function of skeletal muscle in physiology and pathology, and metabolic diseases are associated with glycolytic muscle fibres. Our results showed that ADAR2 KO promoted the conversion of glycolysis muscle fibres to oxidative muscle fibres, suggesting its potentially positive effect on skeletal muscle. But the data apply only to animal sciences research; it is necessary to further study the application of muscle fibre type in human physiology.

At the beginning of this study, we conducted experiments on both male and female mice. Our results showed that ADAR2 KO reduced HFD‐induced body weight gain, liver tissue weight gain and hepatic lipid accumulation in male mice but not in female mice. Indeed, glucose intolerance, IR and elevated fasting plasma glucose levels were improved in male ADAR2 KO mice fed with HFD while not observed in female ADAR2 KO mice fed with HFD. In this study, we aimed to evaluate the possible physiological function of ADAR2 in the association between NAFLD and sarcopenia. Our data suggest that ADAR2 KO improved HFD‐induced NAFLD in male mice but not in female mice. Hence, we focused on male mice for the rest of the studies. However, the underlying mechanisms by which ADAR2 KO improved HFD‐induced NAFLD in male mice but not in female mice, respectively, will have to be clarified in future studies.

In conclusion, we demonstrate that ADAR2 deficiency improves glucose intolerance, IR, hyperlipidaemia, hepatic lipid accumulation and injury. ADAR2 deficiency also improved physical performance and skeletal muscle atrophy. ADAR2 deficiency abolished HFD‐induced SAA1 levels. Increased SAA1 might be involved as a regulatory factor in developing sarcopenia in NAFLD. ADAR2 deficiency may provide a potential therapeutic strategy for NAFLD‐associated sarcopenia.

## Conflict of interest statement

The authors declare no competing interests.

## Supporting information


**Figure S1.** Details of number of animals used in each experiment. (a) Experimental timeline. (b) Experimental procedures and number of animals used in each experiment.
**Figure S2.** Effects of ADAR2 KO on body weight, and organ weight in female obese mice. Physiological parameters in mice from the age of 5 to 25 weeks. (A) Body weight of mice during the feedings. *n* = 18 mice per group. (B) Quantitative results of body weight of mice after the end of regimen. *n* = 18 mice per group. (C) weights of liver, epididymal adipose, epicardial adipose, BAT, and kidney derived from WT and ADAR2 KO mice fed with ND or HFD are shown. *n* = 18 mice per group. All data are expressed as mean ± SEM. Tukey's multiple comparison test after the two‐way ANOVA was conducted for (A)‐(C). *ND‐WT group versus HFD‐WT group or ND‐KO group versus HFD‐KO group; *p < 0.05, *p < 0.01, ***p < 0.001, ****p < 0.0001; n.s, not significant.
**Figure S3.** Effects of ADAR2 KO on food intake, energy intake and water intake in male and female mice. Food intake (male: a, female: d), and energy intake (male: b, female: e) and water intake (male: c, female: f) derived from WT and ADAR2 KO mice fed with ND or HFD are shown. Data were expressed as mean±SEM. Tukey's multiple comparison test after the two‐way ANOVA was conducted for (A)‐(f).
**Figure S4.** Effects of ADAR2 KO on ADAR2 KO on blood glucose levels in female obese mice. (A) Blood glucose levels during IPGTT in male mice (Left panel). Analysis of area under the curve (AUC) of IPGTT results (Right panel). *n* = 10 mice per group. (B) Blood glucose levels during IPITT in male mice (Left panel). Analysis of area under the curve (AUC) of IPGTT results (Right panel). *n* = 10 mice per group. (C) Fasting plasma glucose levels of female mice. *n* = 10 mice per group. All data are expressed as mean ± SEM. Tukey's multiple comparison test after the two‐way ANOVA was conducted for (A)‐(C). *ND‐WT group versus HFD‐WT group or ND‐KO group versus HFD‐KO group; *p < 0.05, **p < 0.01, ***p < 0.001; n.s, not significant.
**Figure S5.** ADAR2 KO affects skeletal muscle fiber type in vivo. The mice were sacrificed, gastrocnemius muscle were collected for experiments. qRT‐PCR analysis of genes associated with regulation of slow‐twitch fibers (MEF2C, and TNNI1) and fast‐twitch fibers (MYH4 and TNNI2), respectively, Results are derived from three independent experiments performed in triplicate. *p < 0.05, ****p < 0.001, vs. ND group, #p < 0.05, ###p < 0.001 vs. HFD‐WT group.
**Figure S6.** Recombinant mouse SAA1 caused primary myotube atrophy. (A) Representative images of immunofluorescence staining with anti‐myosin heavy chain (MyHC) antibody (green). Nuclei were stained with DAPI (blue). Scale bar = 50 μm. (upper panel). Representative quantification of myotube numbers were measured (lower panel). (B) Western blot analysis of the protein expression of myogenic markers (MHC, MyoG) in lysates from primary myoblasts. All data are expressed as mean ± SEM. Unpaired two‐tailed Student's t‐test was conducted for (B). *SAA1‐WT group versus control‐WT group or SAA1‐KO group versus control‐KO group; *p < 0.05, **p < 0.01; n.s, not significant.
**Table S1.** Name and sequences of primers for q‐PCR.
**Table S2.** Antibodies used in this study.
