# Supplementary material for: ADAR2 deficiency ameliorates non‐alcoholic fatty liver disease and muscle atrophy through modulating serum amyloid A1
Source: J Cachexia Sarcopenia Muscle. 2024 Mar 27;15(3):949–62. doi: 10.1002/jcsm.13460 (PMC11154747; doi:10.1002/jcsm.13460)
Supplement: Supplementary file 1 — Figure S1. Details of number of animals used in each experiment. (a) Experimental timeline. (b) Experimental procedures and number of animals used in each experiment. Figure S2. Effects of ADAR2 KO on body weight, and organ weight in female obese mice. Physiological parameters in mice from the age of 5 to 25 weeks. (A) Body weight of mice during the feedings. n = 18 mice per group. (B) Quantitative results of body weight of mice after the end of regimen. n = 18 mice per group. (C) weights of liver, epididymal adipose, epicardial adipose, BAT, and kidney derived from WT and ADAR2 KO mice fed with ND or HFD are shown. n = 18 mice per group. All data are expressed as mean ± SEM. Tukey's multiple comparison test after the two‐way ANOVA was conducted for (A)‐(C). *ND‐WT group versus HFD‐WT group or ND‐KO group versus HFD‐KO group; *p < 0.05, *p < 0.01, ***p < 0.001, ****p < 0.0001; n.s, not significant. Figure S3. Effects of ADAR2 KO on food intake, energy intake and water intake in male and female mice. Food intake (male: a, female: d), and energy intake (male: b, female: e) and water intake (male: c, female: f) derived from WT and ADAR2 KO mice fed with ND or HFD are shown. Data were expressed as mean±SEM. Tukey's multiple comparison test after the two‐way ANOVA was conducted for (A)‐(f). Figure S4. Effects of ADAR2 KO on ADAR2 KO on blood glucose levels in female obese mice. (A) Blood glucose levels during IPGTT in male mice (Left panel). Analysis of area under the curve (AUC) of IPGTT results (Right panel). n = 10 mice per group. (B) Blood glucose levels during IPITT in male mice (Left panel). Analysis of area under the curve (AUC) of IPGTT results (Right panel). n = 10 mice per group. (C) Fasting plasma glucose levels of female mice. n = 10 mice per group. All data are expressed as mean ± SEM. Tukey's multiple comparison test after the two‐way ANOVA was conducted for (A)‐(C). *ND‐WT group versus HFD‐WT group or ND‐KO group versus HFD‐KO group; *p < 0.05, **p < 0.01, [file JCSM-15-949-s001.pdf]

ADAR2 deficiency ameliorates nonalcoholic fatty liver disease and muscle atrophy through modulating serum amyloid A1

†Mei-Lang Kung[1], †Tai-Hua Yang[2,3], Chia-Chi Lin[4], Jia-Yun Ho[4], Tzu-Chi Hung[4], Chih-Hsiang Chang[4], Kuan-Wen Huang[4], Chien-Chin Chen[5,6,7,8], Yun-Wen Chen[4*]

[1]Department of Medical Education and Research, Kaohsiung Veterans General Hospital, Kaohsiung, Taiwan.

[2]Department of Biomedical Engineering, College of Engineering, National Cheng Kung University, Tainan, Taiwan.

[3]Department of Orthopedic Surgery, National Cheng Kung University Hospital, College of Medicine, National Cheng Kung University, Tainan, Taiwan

[4]Department of Pharmacology, College of Medicine, National Cheng Kung University, Tainan, Taiwan

[5]Department of Pathology, Ditmanson Medical Foundation Chia-Yi Christian Hospital, Chiayi, Taiwan;

[6]Department of Cosmetic Science, Chia Nan University of Pharmacy and Science, Tainan, Taiwan;

[7]Ph.D. Program in Translational Medicine, Rong Hsing Research Center for Translational Medicine, National Chung Hsing University, Taichung, Taiwan.

[8]Department of Biotechnology and Bioindustry Sciences, College of Bioscience and Biotechnology, National Cheng Kung University, Tainan, Taiwan.

† These authors contributed equally

*Address correspondence to:

Yun-Wen Chen, PhD,

Department of Pharmacology, College of Medicine, National Cheng Kung University, Tainan 701, Taiwan

Tel: 886-6-2353535 ext 5503; Fax: 886-6-2766185

Suppl. Figure 1

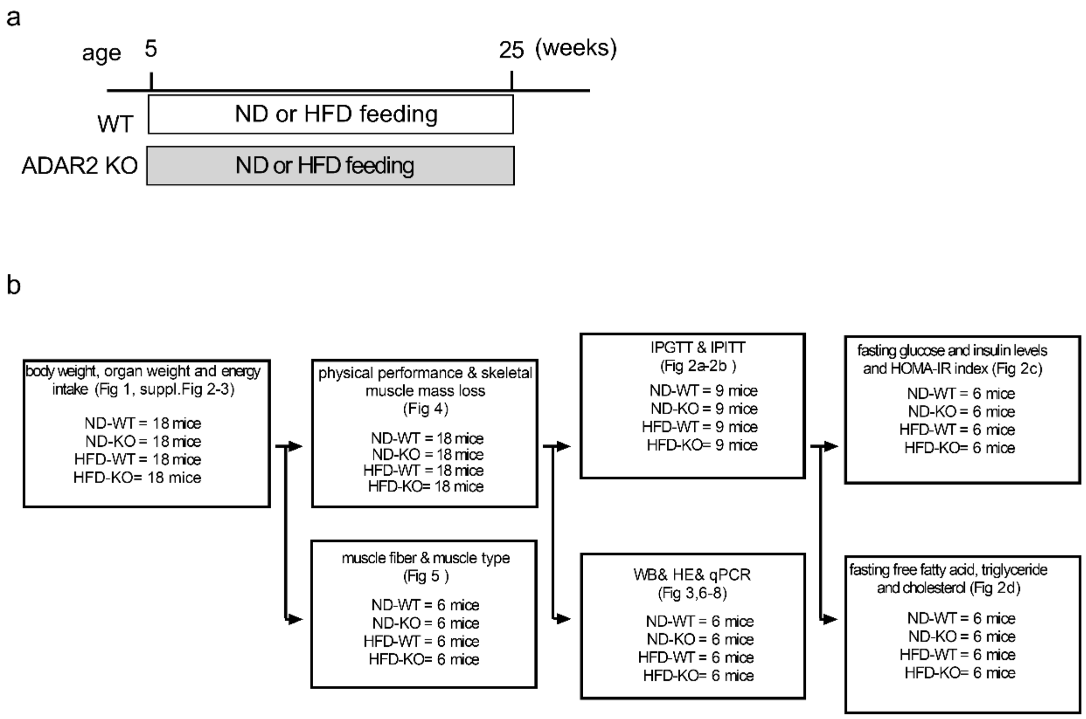

Supplementary Figure S1. Details of number of animals used in each experiment. (a) Experimental timeline. (b) Experimental procedures and number of animals used in each experiment.

Suppl. Figure 2

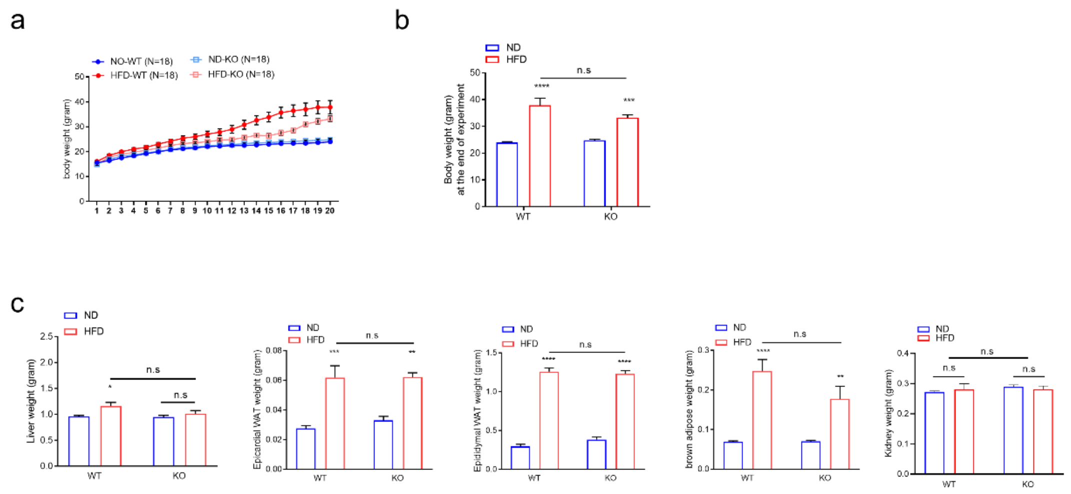

Supplementary Figure S2. Effects of ADAR2 KO on body weight, and organ weight in female obese mice. Physiological parameters in mice from the age of 5 to 25 weeks. (A) Body weight of mice during the feedings. n = 18 mice per group. (B) Quantitative results of body weight of mice after the end of regimen. n = 18 mice per group. (C) weights of liver, epididymal adipose, epicardial adipose, BAT, and kidney derived from WT and ADAR2 KO mice fed with ND or HFD are shown. n=18 mice per group. All data are expressed as mean ± SEM. Tukey's multiple comparison test after the two-way ANOVA was conducted for (A)-(C). *ND-WT group versus HFD-WT group or ND-KO group versus HFD-KO group; *p< 0.05, *p< 0.01, ***p < 0.001, ****p < 0.0001; n.s, not significant.

Suppl. Figure 3

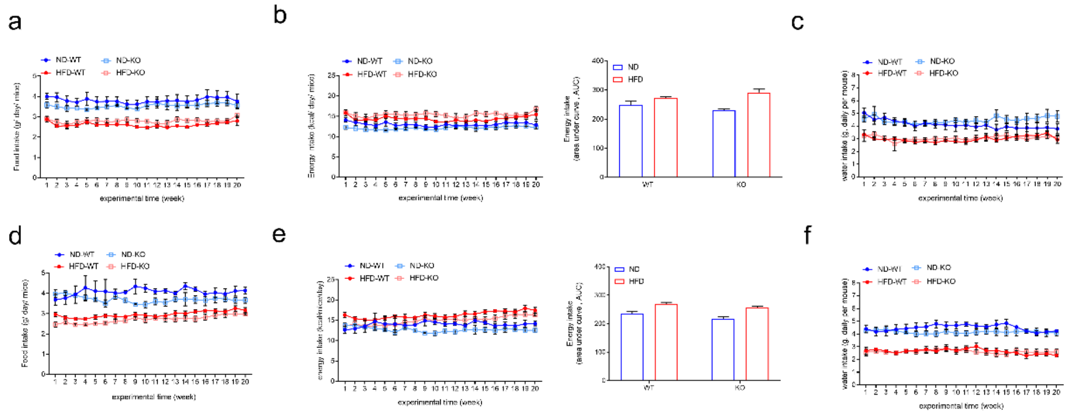

Supplementary Figure S3. Effects of ADAR2 KO on food intake, energy intake and water intake in male and female mice. Food intake (male: a, female: d), and energy intake (male: b, female: e) and water intake (male: c, female: f) derived from WT and ADAR2 KO mice fed with ND or HFD are shown. Data were expressed as mean±SEM. Tukey's multiple comparison test after the two-way ANOVA was conducted for (A)-(f).

Suppl. Figure 4

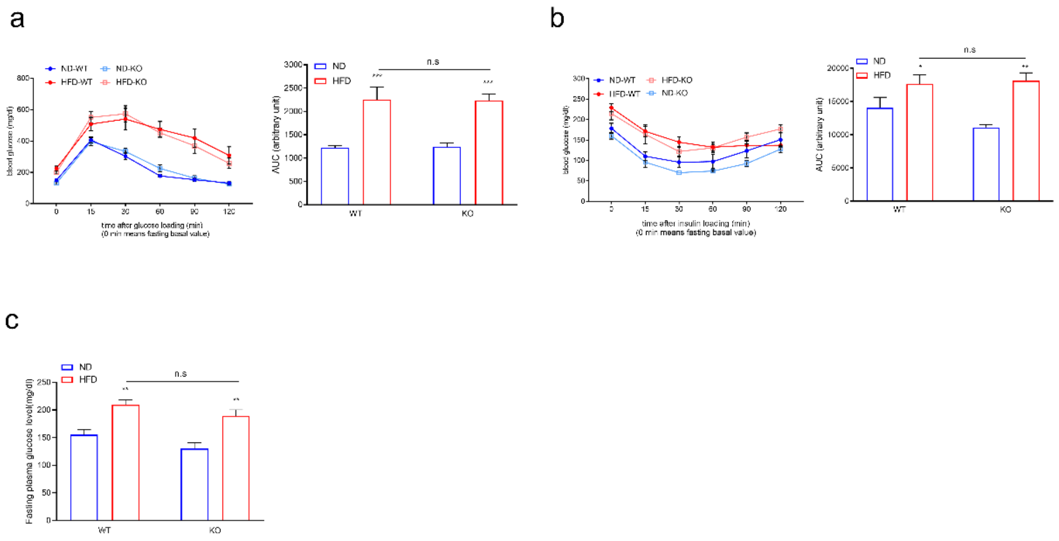

Supplementary Figure 4. Effects of ADAR2 KO on ADAR2 KO on blood glucose levels in female obese mice. (A) Blood glucose levels during IPGTT in male mice (Left panel). Analysis of area under the curve (AUC) of IPGTT results (Right panel). n = 10 mice per group. (B) Blood glucose levels during IPITT in male mice (Left panel). Analysis of area under the curve (AUC) of IPGTT results (Right panel). n = 10 mice per group. (C) Fasting plasma glucose levels of female mice. n=10 mice per group. All data are expressed as mean ± SEM. Tukey's multiple comparison test after the two-way ANOVA was conducted for (A)-(C). *ND-WT group versus HFD-WT group or ND-KO group versus HFD-KO group; *p < 0.05, **p < 0.01, ***p < 0.001; n.s, not significant.

Suppl. Figure 5

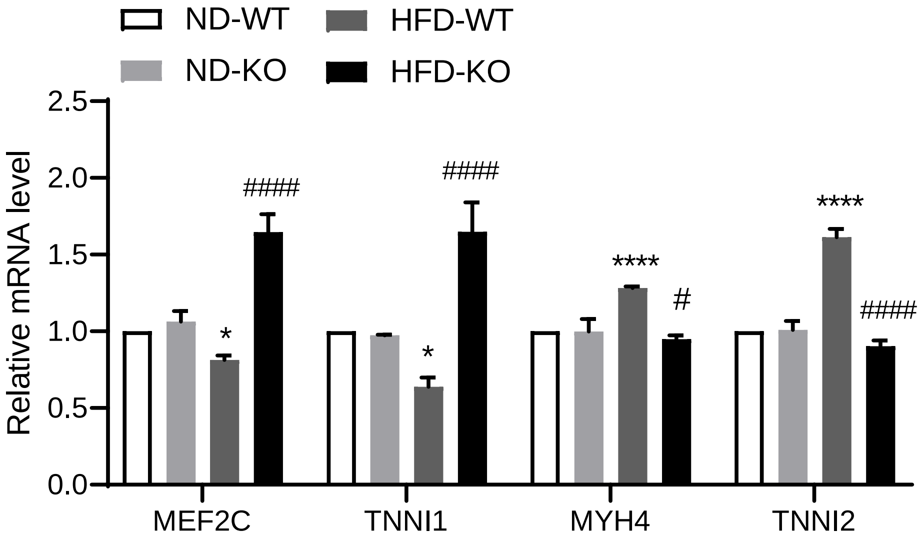

Supplementary Figure S5. ADAR2 KO affects skeletal muscle fiber type in vivo. The mice were sacrificed, gastrocnemius muscle were collected for experiments. qRT-PCR analysis of genes associated with regulation of slow-twitch fibers (MEF2C, and TNNI1) and fast-twitch fibers (MYH4 and TNNI2), respectively, Results are derived from three independent experiments performed in triplicate. *p < 0.05, ****p < 0.001, vs. ND group, #p < 0.05, ###p < 0.001 vs. HFD-WT group.

Suppl. Figure 6

a

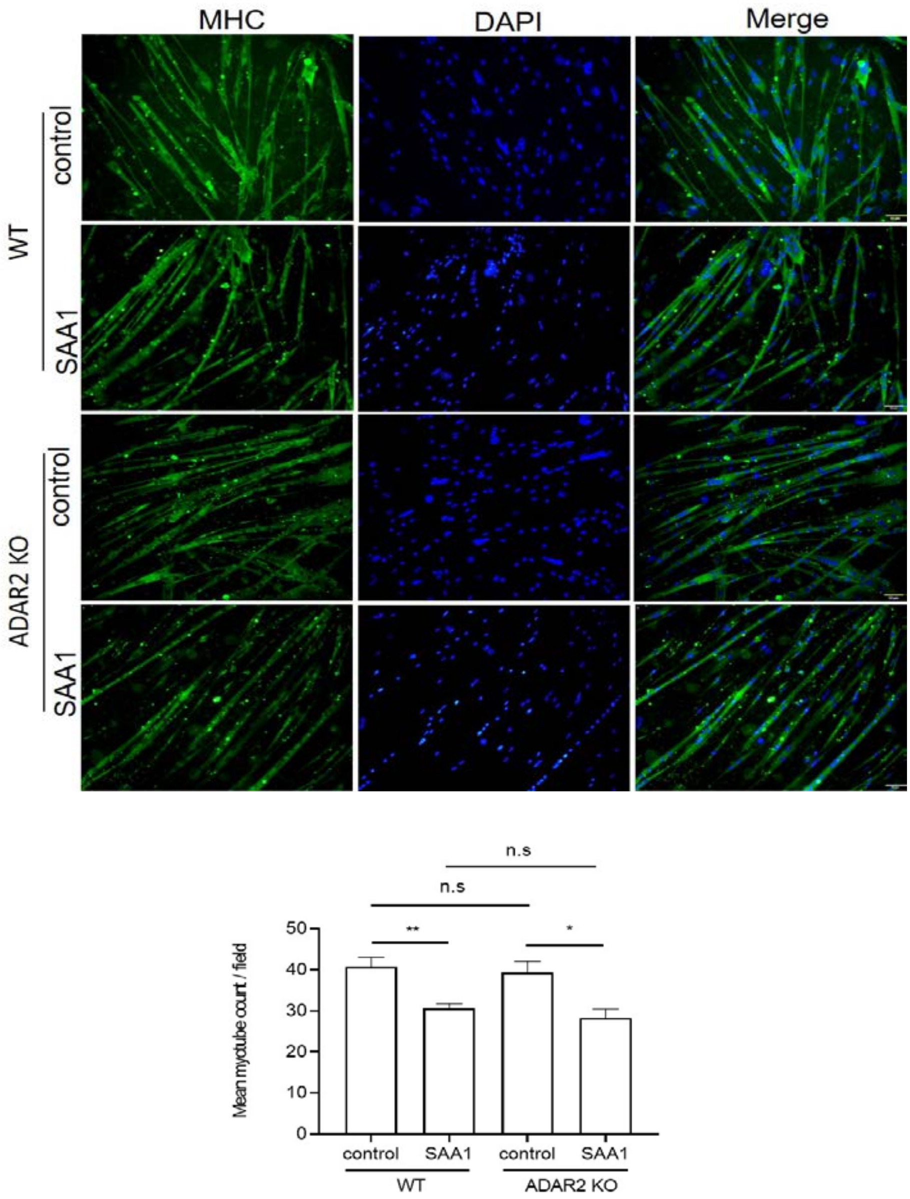

b

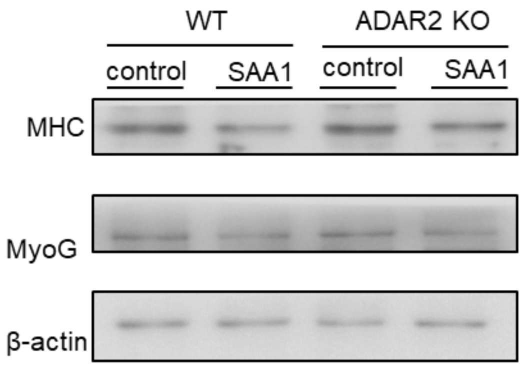

Supplementary Figure S6. Recombinant mouse SAA1 caused primary myotube atrophy. (A) Representative images of immunofluorescence staining with anti-myosin heavy chain (MyHC) antibody (green). Nuclei were stained with DAPI (blue). Scale bar = 50 μm. (upper pannel). Representative quantification of myotube numbers were measured (lower panel). (B) Western blot analysis of the protein expression of myogenic markers (MHC, MyoG) in lysates from primary myoblasts. All data are expressed as mean ± SEM. Unpaired two-tailed Student's t-test was conducted for (B). *SAA1-WT group versus control-WT group or SAA1-KO group versus control-KO group; *$p < 0.05$, **$p < 0.01$; n.s, not significant.

Materials and Methods

Measuring fasting plasma levels of glucose and insulin

After 12-h fasting, the mice were anesthetized and blood samples were collected from the cardiac puncture with heparinized capillary tubes. Plasma was collected after centrifuging the blood at 3000 × g for 10 min. Plasma glucose levels were determined by using a commercial glucose-oxidase kit (Cat. #: 11538, BioSystems, Barcelona, Spain). Plasma insulin levels were determined by using a commercial mouse insulin ELISA kit (Cat. #: 10-1247-01, Mercodia, Uppsala, Sweden).

Calculating homeostasis model assessment insulin resistance index

The homeostasis model assessment insulin resistance (HOMA-IR) index was calculated as followed formula: fasting glucose (mM) × fasting insulin (mU/l)/22.5.

Circulating lipid concentration measurements

The plasma specimens were directly applied to commercial quantification colorimetric kits to detect the selected plasma lipid levels. Commercial quantification colorimetric kits were used to measure the circulating triglycerides (K622-100, BioVision, Milpitas, CA, USA), free fatty acid (K612-100, BioVision), HDL (K613-100, BioVision) and LDL/VLDL (K613-100, BioVision).

Measurement of Circulating high-sensitivity C-reactive protein

The plasma level of high-sensitivity C-reactive protein (hs-CRP) was measured according to the manufacturer’s instructions (Elabscience Biotechnology CO. Ltd., China).

Immunoblotting

Antibodies against AKT (1:1000, Cat. #: 9272), phospho-AKT (1:1000, Cat. #: 4060), FoxO1 (1:1000, Cat. #2880), and phospho-FoxO1 (1:1000, Cat. #9461) were from Cell Signaling Technology. Antibodies against MAFbx (1:800, Cat. # sc-166806), and MuRF1 (1:800, Cat. # sc-398608) were from Santa Cruz Biotechnology. Antibodies against GAPDH (1:5000, Cat. # MA5-15738) were from Invitrogen. Antibodies against MHC (1:5000, Cat. #: 3E8-3D3) were from Developmental Studies Hybridoma Bank (DSHB). Antibodies against myogenin (1:1000, Cat. #: A6664) were from Abclonal. For western blotting, Gastrocnemius muscles of mice were lysed with ice-cold commercial tissue protein extraction reagent (Cat. #: 78510, Thermo Fisher Scientific, Waltham, MA, USA) containing complete

protease inhibitor cocktail tablets (Cat. #: 04693132001, Roche, Basel, Switzerland). Protein concentrations were determined by a Bio-Rad protein assay. An equal amount of protein lysates was separated by SDS-PAGE and then transferred to nitrocellulose membranes. Membranes were blocked in blocking buffer (5% non-fat milk in 1X TBST) and then incubated with primary antibodies, washed, and incubated with the corresponding horseradish peroxidase-conjugated secondary antibodies (Cat. #: 115-035-003 and 111-035-003, Jackson ImmunoResearch, West Grove, PA, USA) and visualized with western blotting luminol reagent (Cat. #: sc-2048, Santa Cruz Biotechnology, Inc.). Bands in the immunoblots were quantified by using ImageQuant LAS 4000 (GE Healthcare, Chicago, IL, USA).

Real-time quantitative PCR analysis

Total RNAs were extracted from gastrocnemius muscles using TRIzol Reagent (Cat. #: 15596018, Ambion, Life Technologies Corporation). The reverse transcription reaction was performed on 2 μg of RNA using SuperScript III First-standard Synthesis System (Cat. #: 18080-051, Invitrogen) with random hexamer primers according to the manufacturer's directions. SYBR Green (Cat. #: 4385612, Thermo Fisher Scientific) was used to quantify the PCR amplification products. The expression levels of the target mRNA were measured using a real-time PCR system (Applied Biosystems, Thermo Fisher Scientific). The target mRNA levels were normalized to the levels of the housekeeping gene β-actin, which was used as the endogenous control. The sequences of the primers are listed in Supplementary Table 1.

Myoblast culture and differentiation.

The murine myoblasts C2C12 cells were cultured in Dulbecco's modified Eagle's medium (DMEM) supplemented with 10% fetal bovine serum (FBS) and 1% penicillin/streptomycin at 37 ℃ in a humidified atmosphere containing 5% CO2. To induce C2C12 myotube differentiation, the medium was replaced by DMEM supplemented with 2% heat-inactivated horse serum (DM) at subconfluent culture and maintained for the time indicated in the corresponding experiments.

Immunostaining of C2C12 myotubes.

C2C12 cells were differentiated on coverslips for 5 days and treated as indicated. Afterward, C2C12 cells were fixed with 4% paraformaldehyde, and treated with 0.1% Triton X-100 to identify permeable cells. After blocking with 3% bovine serum albumin, primary antibodies anti-myosin heavy chain (1:400; Cat. #: 3E8-3D3, DSHB) were added for overnight incubation at 4 °C. The secondary antibody goat anti-mouse Alexa Fluor 488 was then added for incubation in the dark for 1 h, followed by image acquisition by Olympus upright fluorescent microscope (Olympus Corporation, Tokyo, Japan). Cell diameter, length, number, and fusion index were measured using ImageJ software (NIH, Bethesda, MA, USA).

Isolation, culturing and differentiation of primary myoblast from skeletal muscle of adult mice

Isolation of primary myoblast from skeletal muscle of adult mice was performed as previously described (1). The purified myoblasts were cultured in the myoblast growth medium at 37 ℃ in a humidified

atmosphere containing 5% CO_2. To induce primary myoblast differentiation, the medium was replaced by a differentiation medium at subconfluent culture and maintained for the time indicated in the corresponding experiment

Immunostaining of primary myoblast.

Primary myoblasts were differentiated on coverslips for 5 days and treated as indicated. Afterward, Primary myoblasts were fixed with 4% paraformaldehyde, and treated with 0.1% Triton X-100 to identify permeable cells. After blocking with 3% bovine serum albumin, primary antibodies anti-myosin heavy chain (1:400; Cat. #: 3E8-3D3, DSHB) were added for overnight incubation at 4 °C. The secondary antibody goat anti-mouse Alexa Fluor 488 was then added for incubation in the dark for 1 h, followed by image acquisition by Olympus upright fluorescent microscope (Olympus Corporation, Tokyo, Japan). Cell diameter, length, number, and fusion index were measured using ImageJ software (NIH, Bethesda, MA, USA).

References

1. Hindi L, McMillan JD, Afroze D, Hindi SM, Kumar A (2017): Isolation, Culturing, and Differentiation of Primary Myoblasts from Skeletal Muscle of Adult Mice. *Bio Protoc* 7(9):e2248.

Supplementary Table 1 – Name and sequences of primers for q-PCR.

Primer	Sequence
CD36	Forward 5'-GAC TGG GAC CAT TGG TGA TGA-3'<br>Reverse 5'-AAG GCC ATC TCT ACC ATG CC-3'
PPARγ	Forward 5'-TTG ACA CCA TAC TTG AGC AGA-3'<br>Reverse 5'-CAG GAG CAG AGC AAA GAG GT-3'
PPARα	Forward 5'-GTT CAC CCT GAT TCC TGA TGT C-3'<br>Reverse 5'-CCT GCT TCC TGC CAC TTG-3'
CPT1A	Forward 5'- ATC AAT CGG ACT CTG GAA ACG G-3'<br>Reverse 5'-TCA GGG AGT AGC GCA TGG T-3'
SREBP1	Forward 5'- CAC AGG TTC CCC ATA GAC AAA-3'<br>Reverse 5'-GGA GGC AGA GAG CAG AGA TG-3'
ACC	Forward 5'-GGC CAG TGC TAT GCT GAG AT-3'<br>Reverse 5'-AGG GTC AAG TGC TGC TCC A-3'
FAS	Forward 5'-CTG CGG AAA CTT CAG GAA ATG-3'<br>Reverse 5'-GGT TCG GAA TGC TAT CCA GG-3'
SCD1	Forward 5'-TCT TC=C TTA TCA TTG CCA ACA CCA-3'<br>Reverse 5'-GCG TTG AGC ACC AGA GTG TAT CG-3'
TNFα	Forward 5'-TGT GCT CAG AGC AGC TTT CAA CAA C-3'<br>Reverse 5'-GCC CAT TTG AGT CCT TGA TG-3'
IL1-β	Forward 5'-TGAAGT TGA CGG ACC CCA AA-3'<br>Reverse 5'-TTG ATG TGC TGC TGC GAG AT-3'
IL-6	Forward 5'-TTC CAT CCA GTT GCC TTC TTG-3'<br>Reverse 5'-GGG AGT GGT ATC CTC TGT GAA GTC-3'
iNOS	Forward 5'- TGA CGC TCG GAA CTG TAG CAC -3'<br>Reverse 5'- TGA TGG CCG ACC TGA TGT T -3'
IL-12	Forward 5'- GCG GAG CTG CTA CAC TCT CT -3'<br>Reverse 5'- GAC CTG AAC GCA GAA TGT CA -3'
Arg1	Forward 5'- CCA CAG TCT GGC AGT TGG AAG -3'<br>Reverse 5'- GGT TGT CAG GGG AGT GTT GAT G -3'
Fizz1	Forward 5'- CCT GCT GGG ATG ACT GCT A -3'<br>Reverse 5'- TGG GTT CTC CAC CTC TTC AT -3'
Ym1	Forward 5'- GCC ACT GAG GTC TGG GAT GC -3'<br>Reverse 5'- TCC TTG AGC CAC TGA GCC TTC -3'
IL-10	Forward 5'- GGT TGC CAA GCC TTA TCG GA -3'<br>Reverse 5'- ACC TGC TCC ACT GCC TTG CT -3'

SAA1	Forward 5’- GAC ACC ATT GCT GAC CAG GAA -3’<br>Reverse 5’- GGC AGT CCA GGA GGT CTG TAG T -3’
MyHC Ⅰ	Forward 5’- GAATGGCAAGACGGTGACTGTG-3’<br>Reverse 5’- GGAAGCGTAGCGCTCCTTGAG-3’
MyHC Ⅱa	Forward 5’- ATCAACCAGCAGCTGGACACCA-3’<br>Reverse 5’- TCCAGCACGAACATGTGGTGGT-3’
MyHC Ⅱb	Forward 5’- ACAGACTAAAGTGAAAGCCTACAA-3’<br>Reverse 5’- CACATTTTGTGATTTCTCCTGTCAC-3’
MyHC Ⅱx	Forward 5’- CCAATGAAACCAAGACTCCTGG-3’<br>Reverse 5’- TGCTATCGATGAACTGTCCCTC-3’
MEF2C	Forward 5’- CCAATGAAACCAAGACTCCTGG-3’<br>Reverse 5’- GGCGGCATGTTATGTAGGTG-3’
TNNI1	Forward 5’- ATGCCGGAAGTTGAGAGGAA-3’<br>Reverse 5’- CTGAAGGGCACTGAGAGACA-3’
MYH4	Forward 5’- CAATCAGGAACCTTCGGAACAC-3’<br>Reverse 5’- GTCCTGGCCTCTGAGAGCAT-3’
TNNI2	Forward 5’- GATGAGGAGAAGCGCAACAG-3’<br>Reverse 5’- TTTCTCCTCTTCAGCCACGT-3’
GAPDH	Forward 5’-ATG GTG AAG GTC GGT GTG A-3’<br>Reverse 5’-AAT CTC CAC TTT GCC ACT GC-3’

Supplementary Table 2 – Antibodies used in this study.

Name	Catalog number	company
AKT	9272	Cell Signaling
phospho-AKT	4060	Cell Signaling
FoxO1	2880	Cell Signaling
phospho-FoxO1	9461	Cell Signaling
MAFbx	sc-166806	Santa Cruz
MuRF1	sc-398608	Santa Cruz
GAPDH	MA5-15738	Invitrogen.
laminin	L9393	Sigma-aldrich
MHC-I	BA-F8	Developmental Studies Hybridoma Bank
MHC-II	SC-71	Developmental Studies Hybridoma Bank
